# The inferior frontal gyrus and familial risk for bipolar disorder

**DOI:** 10.1093/psyrad/kkac022

**Published:** 2022-12-06

**Authors:** Kun Qin, John A Sweeney, Melissa P DelBello

**Affiliations:** Department of Psychiatry and Behavioral Neuroscience, University of Cincinnati College of Medicine, Cincinnati, OH 45219, USA; Huaxi MR Research Center (HMRRC), Department of Radiology, West China Hospital of Sichuan University, Chengdu, Sichuan 610041, China; Department of Psychiatry and Behavioral Neuroscience, University of Cincinnati College of Medicine, Cincinnati, OH 45219, USA; Huaxi MR Research Center (HMRRC), Department of Radiology, West China Hospital of Sichuan University, Chengdu, Sichuan 610041, China; Department of Psychiatry and Behavioral Neuroscience, University of Cincinnati College of Medicine, Cincinnati, OH 45219, USA

**Keywords:** bipolar disorder, prefrontal, limbic, familial risk, resilience, magnetic resonance imaging, genetic risk

## Abstract

Bipolar disorder (BD) is a familial disorder with high heritability. Genetic factors have been linked to the pathogenesis of BD. Relatives of probands with BD who are at familial risk can exhibit brain abnormalities prior to illness onset. Given its involvement in prefrontal cognitive control and in frontolimbic circuitry that regulates emotional reactivity, the inferior frontal gyrus (IFG) has been a focus of research in studies of BD-related pathology and BD-risk mechanism. In this review, we discuss multimodal neuroimaging findings of the IFG based on studies comparing at-risk relatives and low-risk controls. Review of these studies in at-risk cases suggests the presence of both risk and resilience markers related to the IFG. At-risk individuals exhibited larger gray matter volume and increased functional activities in IFG compared with low-risk controls, which might result from an adaptive brain compensation to support emotion regulation as an aspect of psychological resilience. Functional connectivity between IFG and downstream limbic or striatal areas was typically decreased in at-risk individuals relative to controls, which could contribute to risk-related problems of cognitive and emotional control. Large-scale and longitudinal investigations on at-risk individuals will further elucidate the role of IFG and other brain regions in relation to familial risk for BD, and together guide identification of at-risk individuals for primary prevention.

## Introduction

Bipolar disorder (BD) is one of the most common and debilitating mental illnesses characterized by recurrent manic episodes and typically also depressive episodes, and is a leading cause of social and economic challenges worldwide (Whiteford *et al*., 2013). BD is known to be a familial disorder (Craddock & Sklar, 2013), and studies of individuals having a first-degree relative with BD can be informative about biological risk factors and potentially primary prevention (Gershon *et al*., 2022). Establishing quantitative biomarkers for various illness-related traits are a promising strategy for understanding the pathophysiological mechanisms associated with both risk and resilience.

The emergence of psychoradiology as a field advancing the clinical application of neuroimaging in psychiatry enables the quantitative characterization of subtle alterations in brain systems that are not revealed by routine clinical visual inspection (Lui *et al*., 2016). With the development of advanced imaging and analytic techniques, exploring neuroimaging biomarkers in various psychiatric conditions has become increasingly valuable over the past two decades (Aydin*et al*., 2019). Substantial neuroimaging studies have reported extensive brain structural and functional abnormalities in BD patients (Ching *et al*., 2022). Although findings are not consistent across studies due to clinical and methodological heterogeneity, abnormalities within the frontolimbic circuitry are believed to represent well-established neurobiological underpinnings of mood symptoms in BD (Lui *et al*., 2015; Passarotti *et al*., 2011; Vai *et al*., 2019). In particular, aberrant interactions between prefrontal inhibitory and limbic affective systems have been shown to play a key role in the disrupted emotion regulation in BD patients.

Genetic factors cannot be neglected in the development and pathogenesis of BD (Craddock & Sklar, 2013; Lee *et al*., 2013). As a familial disorder, the heritability of BD is estimated to be 75% in first-degree relatives (Craddock & Sklar, 2013). Neuroimaging studies of at-risk first-degree relatives of probands with BD provide an approach for investigating neural mechanisms of familial risk for BD. Of note, even at-risk individuals who remain psychiatrically well can still show brain structural and functional abnormalities, suggesting that risk and resilience factors may contribute to illness onset (Wiggins *et al*., 2017). Although alterations of several brain regions have been found to be associated with familial risk for BD in the past decade, the inferior frontal gyrus (IFG) has been one of the most replicable findings in at-risk individuals (Cattarinussi *et al*., 2019; Zhang *et al*., 2020). The IFG plays an important role in multiple cognitive and emotional functions, which may account for its involvement in the pathophysiology of BD (Phillips & Swartz, 2014). Additionally, the structural and functional features of IFG are known to be highly heritable (Mullins *et al*., 2021), consistent with its potential role as a strongest candidate marker of familial risk for BD. Thus, in this review, we summarize multimodal IFG abnormalities in magnetic resonance imaging studies of individuals at familial risk for BD, discuss associated risk and resilience markers, and raise implications for future studies of BD familial risk.

### IFG and familial risk for BD

The prefrontal cortex (PFC) is well organized as a hub supporting cognitive control, scheduling, and optimizing processes implemented by posterior cortical and subcortical regions (Miller & Cohen, 2001). The IFG in humans (often referred to as ventrolateral prefrontal cortex (VLPFC) in brain imaging studies), extending posteriorly to include pars opercularis (Brodmann Area 44), pars triangularis (Brodmann Area 45), and pars orbitalis (Brodmann Area 47/12) (Petrides & Pandya, 2002), is one of the core components of the PFC. Lesion studies have demonstrated that damage to the right IFG disrupts inhibitory control (Iversen & Mishkin, 1970), leading to impaired response inhibition and task switching (Logan *et al*., 2014; Vandierendonck *et al*., 2010). For patients with BD, inhibition is critical for effective emotion regulation, as exaggerated and persistent emotional reactions are a common clinical characteristic of the disorder. Successfully inhibiting attention to emotionally relevant content, especially negative emotional stimuli, can reduce emotion-related impulsivity and facilitate reappraisal (Bartholomew *et al*., 2021; Passarotti *et al*., 2013). The left IFG plays a crucial role in various aspects of language functions including speech production, and semantic and syntactic processing (Gabrieli *et al*., 1998; Hagoort, 2005). Altered language structure has been consistently shown in linguistic studies of BD patients (Raucher-Chéné *et al*., 2017; Weiner *et al*., 2019), including alterations of verbal fluency, semantic content, and speed of language. In summary, abnormalities of the IFG may be important contributors to cognitive deficits in BD (i.e. inhibitory control and language ability), leading to various relevant disturbances of behaviors, such as mood regulation, verbal fluency, cognitive flexibility, and reward processing. However, few studies investigate the role of IFG subregions in the pathogenesis of BD, and future studies are warranted to reveal shared and distinct function of each IFG subregion.

Recent evidence indicates that youth at increased genetic risk for BD initially display impaired verbal ability and affective response inhibition prior to a broad range of neuropsychological dysfunctions (McCormack *et al*., 2016), which is consistent with the forementioned function of IFG. Moreover, according to the clinical staging model of BD, non-mood disorders, such as anxiety disorder, conduct disorder, attention-deficit/hyperactivity disorder, and substance use disorder emerged at an early stage of psychopathological development in at-risk individuals (Raouna *et al*., 2018). Language dysfunction and impulsivity are the common features of these non-mood disorders. Thus, both clinical and neuropsychological evidence support the view that abnormalities of the IFG may be one of the early brain alterations in relatives at genetic risk for BD (Hill *et al*., 2013). Investigating abnormalities associated with the IFG may contribute to the characterization of targets for primary prevention in at-risk individuals.

### Structural abnormalities of the IFG

As revealed by meta-analyses and numerous original reports (Cattarinussi *et al*., 2019; Cattarinussi *et al*., 2022; Sarıçiçek *et al*., 2015; Zhang *et al*., 2020), increased volume in the IFG has become one of the most replicable neuroanatomical signature in individuals at genetic risk for BD. Data on 1558 young students suggested polygenic risk score of BD is associated with greater gray matter volume in the right IFG (Takeuchi *et al*., 2021). A two-center replication-design study demonstrated that unaffected relatives of patients with BD showed a larger right IFG volume (Hajek *et al*., 2013). Another prospective longitudinal study found that high-risk twins had consistently larger IFG volumes compared to low-risk twins throughout a 7-year follow-up (Macoveanu *et al*., 2018). These findings reflected the robustness of IFG enlargement in at-risk individuals, and a close relationship between increased familial risk and enlarged IFG. Longitudinal studies identified an accelerated pattern of IFG volume reduction in at-risk individuals compared to low-risk individuals (Roberts *et al*., 2022). The brain structural trajectory of initial IFG volume increase followed by accelerated decline may thus be a promising indicator of illness development in at-risk individuals. Increased IFG volume at early stage may be an adaptive protection that compensates for progressive IFG volume reduction (Pavuluri *et al*., 2009). Notably, increased right IFG volume in at-risk individuals seems to be more consistent than similar effects in the left hemisphere (Cattarinussi *et al*., 2019; Zhang *et al*., 2020). One possible explanation may be the greater role of the right hemisphere in automatic emotion processing (Wyczesany *et al*., 2018). Since the region of inhibitory control of affective responses is mainly located in the right IFG and the left IFG is more related to language function (Aron *et al*., 2004; Costafreda *et al*., 2006), increased right IFG volume may be related to impairment of affective response modulation, which could be directly related to vulnerability for developing mood disorders. Whether such structural alteration accounts for primary disruption of emotion regulation systems, or a compensatory adjustment to better modulate hyper-reactivity in limbic cortex remains to be determined.

Regional neocortical volume is a product composed of cortical thickness and surface area, which are genetically independent (Grasby *et al*., 2020; Panizzon *et al*., 2009). Drobinin *et al*. identified increased surface area in the pars triangularis of the right IFG in at-risk individuals (Drobinin *et al*., 2019), suggesting an enlarged right IFG is influenced by an increase in surface area. For the left IFG, although volume increase is not consistently observed, reduced cortical thickness has been widely reported. A multicenter study found thinner pars opercularis of the left IFG in high-risk participants compared to low-risk participants (Mikolas *et al*., 2021). Roberts *et al*. found decreased cortical thickness in the pars orbitalis of the left IFG in at-risk individuals (Roberts *et al*., 2016). Papmayer and colleagues found that progressive cortical thinning in the left IFG is related to an increased risk for mood disorder (Papmeyer *et al*., 2015). These findings indicate that left IFG in at-risk individuals may tend to have reduced cortical thickness. Hence, available evidence indicates that the left and right IFG in at-risk individuals have different pathologies, with the left IFG showing cortical thickness changes and right one showing surface area alterations. Given distinct genetic effects on surface area and cortical thickness, left and right IFG may be associated with different genetic risk mechanisms. Furthermore, because surface area alterations are primarily neurodevelopmental in nature, genetic effects may predominate in the left IFG while adaptive compensatory effects during neurodevelopment may be more relevant for alterations in the right hemisphere. Future studies to reveal common and distinct genes associated with specific morphometric abnormalities of the bilateral IFG are warranted.

In addition to regional aberrations, structural disconnection of the IFG has also been identified in at-risk individuals (Roberts *et al*., 2018). Uncinate fasciculus (UF) that connects VLPFC with the amygdala, and has been one of the most consistent findings in studies of white matter tracts in BD. The integrity of UF contributes to positive emotion processing and successful mood regulation. Patients with BD typically showed decreased fractional anisotropy (FA) and increased mean diffusivity (i.e. measures that can reflect fiber integrity) of the UF compared to HC. For BD familial risk, Foley *et al*.found that unaffected siblings of patients irrespective of BD subtype showed lower FA of the UF than HC (Foley *et al*., 2018), and reduced FA in the UF has been significantly correlated with increased risk-taking during gambling task (Linke *et al*., 2013) in at-risk individuals. Roybal *et al*.identified increased FA of the bilateral UF in youth at familial risk for BD (Roybal *et al*., 2015). Li *et al*. did not detect significant group differences in FA or mean diffusivity of the UF, but FA of the right UF could predicted the onset of BD in at-risk individuals during a 6-year follow-up (Li *et al*., 2021). While studies of UF microstructural changes in at-risk individuals are promising, sample sizes are often small. Studies with larger sample sizes in the future will help establish and validate these preliminary findings.

### Functional abnormalities of the IFG

Individuals at familial risk for BD also exhibit functional abnormalities in the IFG. Consistent with increased gray matter volume in the IFG, elevated amplitude of low-frequency fluctuations signals in the IFG has been identified in at-risk individuals (Lin *et al*., 2018), suggesting enhanced spontaneous activity in the IFG during resting state. Increased amplitude of low-frequency fluctuations signal of the IFG, coupled with increased gray matter volume, are consistent with the idea that this region is involved in adaptive compensatory processes to maintain normal inhibitory regulation of limbic reactivity, as increased function may lead to an expansion of neuropil in cortex to increase its volume. Abnormal resting-state functional connectivity of the IFG is also observed in at-risk individuals. Roberts and colleagues found reduced connectivity of the IFG with limbic and striatal areas in at-risk individuals, including the insula, medial cingulate gyrus, and lentiform nucleus (Roberts *et al*., 2017). Singh *et al*.found decreased connectivity between the IFG and caudate in the healthy offspring of parents with BD (Singh *et al*., 2014). Connectivity between the IFG and limbic/striatal areas was also found to be correlated with person-level risk score and mood liability in youth at familial risk for BD (Hafeman *et al*., 2019). Therefore, at-risk individuals typically exhibit a robust pattern of reduced frontolimbic and frontostriatal connectivity. Disconnection of the inhibitory hub (i.e. IFG) from downstream sites of regulation and interoception may lead to the difficulty of inhibition in response to emotional stimuli leading to emotional-related impulsivity seen in at-risk individuals (Ethridge *et al*., 2014; Hill *et al*., 2013). In addition, a clinical trial found that high-risk youths had increased connectivity between IFG and the anterior default mode network following 4 months of family-focused therapy (Singh *et al*., 2021), suggesting connectivity of IFG can further reflect treatment effects and may serve as promising neuromodulation targets for early intervention.

Current knowledge implicates that BD is associated with behavioral dysfunction in multiple emotional and cognitive domains, such as emotional processing, attentional control, behavioral impulse control, working memory, and reward processing (Phillips & Swartz, 2014). Therefore, task-based functional magnetic resonance imaging studies mostly used emotional or cognitive tasks focused on these processes and examined corresponding brain activities in at-risk relatives. Previous studies found that at-risk individuals showed decreased activation in the IFG during explicit emotion processing and increased IFG activation during implicit emotion processing (Brotman *et al*., 2014; Chang *et al*., 2017). Reduced activation of the IFG was identified in at-risk individuals when inhibiting response to emotional cues (Roberts *et al*., 2013). Unaffected offspring of parents with BD exhibited greater VLPFC activity during an emotional working memory paradigm (Ladouceur *et al*., 2013). Kim *et al*. found that at-risk youths showed hyperactivation in the VLPFC during a task-switching paradigm (Kim *et al*., 2012). Although findings were mixed in various cognitive paradigms, a recent neuroimaging meta-analysis robustly identified hyperactivation of the right IFG in at-risk relatives across a range of cognitive tasks (Cattarinussi *et al*., 2019), suggesting a shared pattern of IFG hyperactivation involved in diverse cognitive processes. Regarding the reward processing tasks, previous studies have found greater IFG activation in offspring of parents with BD during reward processing (Singh *et al*., 2014). Given the complexity and heterogeneity involving analytical techniques, task design, and small samples, future studies with large cohorts should be performed to identify robust patterns of IFG activity during specific types of tasks in individuals at familial risk for BD. Further, determining whether such alterations confer increased risk for later onset of BD will be another important avenue of research.

### Risk and resilience markers for BD related to IFG

**Figure 1:**
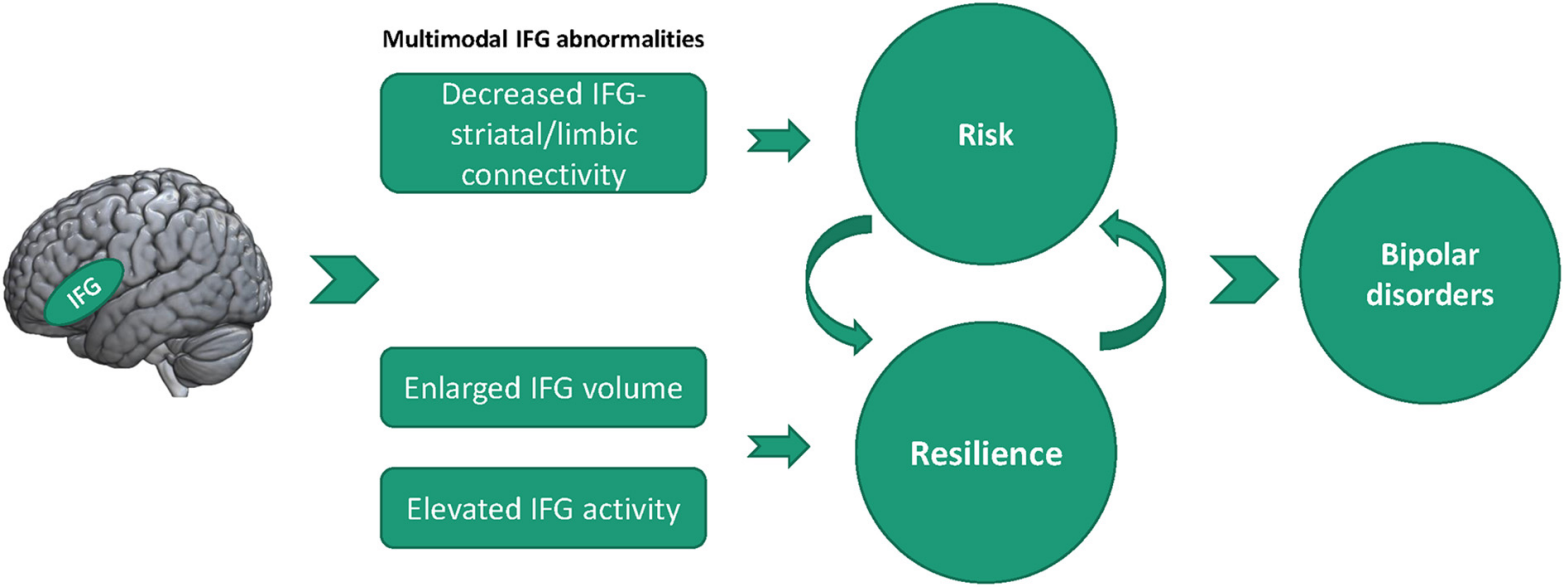
Multimodal IFG abnormalities related to risk and resilience markers for BD. At-risk individuals showed larger gray matter volume and increased functional activities in IFG compared with low-risk controls, which might result from an adaptive brain compensation to support emotion regulation as an aspect of psychological resilience. Connectivity between IFG and downstream limbic or striatal areas was typically decreased in at-risk individuals relative to controls, which might be associated with risk-related problems of cognitive and emotional control.

Figure 1 summarizes the multimodal IFG abnormalities related to risk and resilience markers for BD. In the past decade, numerous neuroimaging studies have made efforts to identify risk endophenotypes for BD. Endophenotypes are intermediate phenotypes that causally connect upstream genes and downstream disease (Gottesman & Gould, 2003). The endophenotypes are illness-relevant, state independent, heritable, and biologically plausible (Gottesman & Gould, 2003). Since an endophenotype in probands can be more frequently found in their unaffected relatives than in the general population, searching for brain abnormalities that are coexpressed in patients and unaffected relatives provides an approach to identify novel risk endophenotypes for BD (Hasler *et al*., 2006). In addition to these risk markers, there are also brain features associated with resilience that may adaptively compensate for the adverse effects of disease vulnerability and expression. In this context, resilience markers are neural features that reduce rather than increase risk for illness. Thus, brain areas where unaffected at-risk individuals exhibit alterations relative to HC, but patients with BD do not, may represent resilience markers. Cross-sectional comparison can only preliminarily suggest brain abnormalities related to risk and resilience. Models of brain circuitry and the nature of the observed alterations can help evaluate the biological plausibility of how markers are related to illness. Longitudinal studies and detailed examination of psychological correlates of alterations are also needed to differentiate how alterations see in at risk individuals are related to illness onset. Further, as risk and resilience effects are always in dynamic balance, determining responses of brain systems to external stressors, such as whether there are positive or negative associations between brain alterations over time, may also help separate risk and resilience markers.

At present, alterations in IFG are not yet established as risk endophenotypes for BD. One set of issues is whether alterations are replicable and consistent regardless of state of illness. Long *et al*.performed a comparative meta-analysis to explore shared brain structural abnormalities between first-episode patients and unaffected first-degree relatives throughout the brain, and concluded that no specific regional volume alterations can represent risk endophenotypes for BD (J. Long *et al*., 2022). Besides, IFG volume seems to change after onset. A smaller IFG volume was identified in patients with BD (Haldane *et al*., 2008; López-Larson *et al*., 2002; Lyoo *et al*., 2004; Stanfield *et al*., 2009). Negative association between the IFG volume and illness duration has been documented in previous reports (Ekman *et al*., 2010; Lyoo *et al*., 2004; Stanfield *et al*., 2009). One potential explanation for these findings is that the initial increased IFG volume in healthy at-risk individuals may reflect compensatory effects to reduce downstream limbic activities. Decreased IFG volume in those who develop BD may result from a loss of such effects. It is also possible that illness onset leads to treatment administration that reduces limbic activity requiring less top-down modulation and thus leads to an associated reduction in IFG volume. When regional volume decomposes into morphological elements including surface area and cortical thickness, we noted that increased surface area in the pars triangularis of the IFG can be observed in both patients and at-risk individuals (Drobinin *et al*., 2019; Woo *et al*., 2021; Yalin *et al*., 2019). Thus, among major gray matter measures (i.e. volume, cortical thickness, surface area), only the surface area of the IFG can become a potential risk endophenotype for BD. Although the IFG volume is heritable (Winkler *et al*., 2010), a genetically and developmentally distinct pathway between surface area and cortical thickness may lead to the inconsistency in representation of risk endophenotype for BD (Panizzon *et al*., 2009).

Familial effects can explain 71–80% of the variance in the UF microstructure (Kochunov *et al*., 2010). Although microstructure of this white matter tract connecting VLPFC and amygdala is highly heritable, whether microstructural alterations of UF coexpress in BD patients and at-risk relatives independent of clinical stages is controversial. Mixed microstructural changes in the UF have been reported in BD patients and at-risk relatives (Emsell *et al*., 2014; Foley *et al*., 2018; Hu *et al*., 2020; Sarıçiçek *et al*., 2016). Meta- and mega-analytic evidence indicate that BD patients have lower FA of the UF compared to HC (Favre *et al*., 2019; E. Xu *et al*., 2022), while similar findings have not been observed in studies of at-risk relatives according to current available meta-analyses (E. Xu *et al*., 2022; M. Xu *et al*., 2022). By using a conjunction meta-analytic approach, shared microstructural changes in the UF between BD patients and at-risk individuals were also not observed (Hu *et al*., 2020). Thus, the microstructural changes in the UF may not represent a risk endophenotype for BD. However, cumulative risk imposed by multiple genetic risk factors varies by individual, with some carrying more risk than others. Whether alterations are seen in those with higher polygenic risk scores remains to be established. Furthermore, given the limited number of studies of at-risk individuals relative to studies of affected individuals, this conclusion may be viewed as preliminary. Future large-scale and longitudinal investigation are required to further verify robust changes in the UF in at-risk individuals.

With regard to the functional changes in the IFG, disrupted frontolimbic (especially connections from VLPFC) and frontostriatal (VLPFC and orbitofrontal cortex) connectivity in BD patients has been widely documented (Phillips & Swartz, 2014). Similar deficits have also been identified in at-risk individuals, suggesting a potential risk endophenotype. For example, Singh *et al*.reported decreased VLPFC-caudate connectivity in healthy offspring of parents with BD showed compared to HCs (Singh *et al*., 2014). Roberts *et al*. found at-risk individuals exhibited disconnection of the IFG from insula and cingulate gyrus (Roberts *et al*., 2017). However, the number of resting-state functional connectivity studies of at-risk individuals is small, and relevant findings have not been well replicated. To fully understand the connectivity-level deficits as trait of risk or resilience for BD, studies with large samples of at-risk individuals at various developmental stages will be informative and more convincingly establish the robustness of these alterations and their clinical relevance.

Studies on functional activation of the IFG during task performance have been performed, but interpretation of these findings is complicated considering the various behavioral paradigms that have been used. Herein, we focus discussion on three of the most widely studied domains (i.e. emotion, reward processing, cognition) in both studies of patients and at-risk relatives. For emotional processing, hypoactivation in the IFG is commonly observed in BD patients when viewing emotional stimuli, especially negative emotional stimuli (Delvecchio *et al*., 2012; Houenou *et al*., 2011). However, current meta-analysis did not reveal robustly reduced activation of the IFG in at-risk individuals during performance of emotional processing tasks (Cattarinussi *et al*., 2019). Nimarko and colleagues performed a 3-year retrospective investigation that compared at-risk youths who converted to a psychiatric diagnosis with those who remained healthy without any psychopathology (Nimarko *et al*., 2019). They found that a “resilience” group exhibited greater deactivation in the IFG than the “converter” group, suggesting the IFG may serve as resilience marker in BD. Wiggins *et al*.also proposed that IFG functional patterns may represent resilience markers by comparing alterations in BD patients and at-risk individuals (Wiggins *et al*., 2017). As there are only few studies indicating hypoactivation in the IFG as risk endophenotype (Brotman *et al*., 2014), findings need to be further verified and replicated.

During reward anticipation, BD patients have been reported to show decreased activation in the IFG compared to HC (X. Long *et al*., 2022). Current reward processing studies of at-risk relatives did not reach consistency in terms of the specific location of abnormalities. However, we noted that the hyperactivation pattern in prefrontal regions is consistently observed based on the limited studies (Linke *et al*., 2012; Manelis *et al*., 2016; Singh *et al*., 2014). Since the direction of activation change in the IFG may be in opposite directions in patients and at-risk relatives, abnormal IFG activation is less likely to be a risk endophenotype but more likely to underlie adaptive protection mechanism in BD.

A meta-analysis of at-risk relatives in studies of task-based cognitive activation protocols included studies of 19 different cognitive tasks. Generally, studies indicated an increased activation in the IFG during task performance (Cattarinussi *et al*., 2019). Similar hyperactivation of the IFG has been reported in BD patients, especially when performing tasks requiring attentional engagement (Lee *et al*., 2019). Thus, greater activation in the IFG during cognitive processing is another promising candidate risk endophenotype. However, work is needed to determine the most useful cognitive paradigms to use for this purpose (e.g. working memory, response inhibition, verbal ability, attention, cognitive flexibility), and potentially to evaluate how emotional activation affects activation profiles seen during cognitive task performance (Schenkel *et al*., 2012). As disruption of cognitive processes during periods of emotional activation has been observed in bipolar patients, evaluating functional IFG abnormalities with and without emotional interference and its impact on cognition is therefore of great importance.

### Future implications on BD-risk study

Previous findings reveal multiple deficiencies and raise new questions and avenues for neuroimaging research on BD risk. First, healthy at-risk individuals show more subtle brain alterations than those who have developed BD,so sufficient sample size in adequately powered studies is critical to detect risk and resilience alterations at an early stage. Second, the relation between the types of alteration seen in patients may vary with those seen in their relative proband. Third, level of polygenic risk may determine the severity of brain system alterations. Fourth, the relation of functional and anatomic changes is not yet well understood. Fifth, the differentiation of risk and resilience changes and, because of compensatory and pathological changes, will require considerable future work but may guide development of causal models of illness risk and etiology. Sixth, the behavioral correlations of brain alterations are not well understood. Seventh, whether different brain alterations in IFG and other brain regions predict later onset of BD is something that needs to be addressed in longitudinal studies of high-risk samples. Last, establishing the developmental trajectory of alterations from youth through the age of high risk for illness onset may provide novel insights into risk mechanisms for BD. Whether risk factors are static or evolve in relation to near-term illness onset is important to know for designing illness prevention strategies.

Another related line of work is to investigate the relation of brain alterations to subclinical features of BD, such as heightened emotional reactivity, moodiness, irritability, etc. One classic way to address this question is to develop and use a clinical staging model of BD to subgroup at-risk individuals. For example, individuals might be grouped into healthy (stage 0), nonmood disorders including personality disorders (stage 1), minor mood disorder (stage 2), major mood disorder (stage 3), and onset of first mania/hypomania episode (stage 4) (Raouna *et al*., 2018). Another approach is to subgroup at-risk individuals according to comorbid psychopathology, such as attention deficit hyperactivity disorder (ADHD), substance use disorder, anxiety, and depression (Lau *et al*., 2018). For example, youth at familial risk for BD often meet DSM-5 diagnostic criteria for ADHD (Axelson *et al*., 2015; Duffy *et al*., 2007), and are initially prescribed psychostimulant treatment (i.e. standard treatment for ADHD) for inattention/hyperimpulsivity symptoms (Greenhill *et al*., 2002). However, studies have reported that psychostimulant exposure can exacerbate mood and possibly accelerate the onset of mania in youth at risk for BD (Carlson *et al*., 2000; DelBello *et al*., 2001). Exploring the difference in neurobiological underpinnings of ADHD between youths with and without familial risk for BD may thus assist in effective and safe treatment selection.

In addition, it is also important to recognize that the development of BD in at-risk individuals is a dynamic interaction between risk and resilience. Tracking the longitudinal trajectory of brain imaging markers of risk and resilience across the course of development and in the epoch before the emergence of BD may provide crucial information about the emergence of illness, and how the balance of risk and resilience factors may determine illness onset. Since the onset of BD often occurs during adolescence, how the brain changes developmentally in association with familial risk is also of interest. Considering this factor may improve more precise identification of risk status in youth at early stage for illness prevention. Environmental effects also play a key role in the pathogenesis of BD (Geoffroy *et al*., 2013), and adverse events including social isolation, childhood trauma, and family poverty, are highly associated with the risk for developing affective disorders (Palmier-Claus *et al*., 2016). In this context, discordant twin studies offer an exclusive opportunity to explore and disentangle the interactive effects of gene and environment on brain given their shared genetic and environmental features (Fagnani *et al*., 2014). Therefore, the fine mapping of brain abnormalities associated with familial risk requires further progress on longitudinal trajectory with interactive brain aging and environmental effects considered.

Moreover, advanced imaging analytic techniques should be applied in BD-risk studies. The human brain is suggested to be a highly connected network that facilitates information processing and passing (Glasser *et al*., 2016). Mental disorders have been better understood as a disruption of the brain connectome instead of resulting from regional abnormality (Rubinov & Bullmore, 2013). Advanced graph theoretical analysis provides an approach to measure global and local network disruptions that have been widely used for various psychiatric illnesses (Bullmore & Sporns, 2009; Bullmore & Bassett, 2011). Up to now, we know of one study using graph theoretical analysis to reveal functional network deficits in youths at genetic risk for BD (Roberts *et al*., 2017). In addition, two studies investigated structural and functional network topological alterations in at-risk youths following systematic mindfulness-based cognitive treatment (Qin *et al*., 2021; Yang *et al*., 2021), suggesting network-level profiles are associated with early interventional effects. Brain network topology is also heritable (Fornito *et al*., 2011; Glahn *et al*., 2010), and identifying genes that contribute to variation in brain structural and functional networks can bridge the gap between connectome and transcriptome, linking gene function to neural phenotypes (Fornito *et al*., 2019). By using partial least squares regression, researchers can test for correlations between spatial topological abnormalities in BD patients or at-risk individuals and spatial gene expression pattern. Lei *et al*.found that genes showing specific expression in excitatory and inhibitory neurons were associated with deficits of morphometric similarity network in BD patients (W. Lei *et al*., 2022). Future studies based on larger samples are warranted to verify network-level abnormalities, as well as associated gene, molecular, and cell function in at-risk individuals.

The ultimate goal of precision psychiatry is to achieve personalization on diagnosis, assessment, and treatment (Bzdok & Meyer-Lindenberg, 2018). Machine learning can be effectively implemented to characterize patients under psychiatric conditions and identify related complex neural patterns in imaging features in a multivariate manner. There have been a few studies applying machine learning to discriminate between low-risk and high-risk individuals based on various imaging modalities (Frangou *et al*., 2017; Hajek *et al*., 2015; Roberts *et al*., 2017). Although these studies revealed individual-level vulnerability and resilience markers, the utility for neuroimaging findings in predicting illness conversion in at-risk youths is more concerned with clinical settings. Thus, future machine learning studies should focus on longitudinal design and predict the emergence of BD or other psychopathology during follow-up in healthy at-risk individuals. Furthermore, some novel machine learning models such as graph neural networks can capture important network-level information (D. Lei *et al*., 2022; Qin *et al*., 2022), and multidimensional features beyond imaging (e.g. clinical, behavioral, demographic, and genomic data) can be used for model training to improve prediction accuracies.

## Conclusions

This review shows that structural and functional abnormalities in the IFG are correlated with genetic risk for BD. By comparing high-risk relatives of BD patients with low-risk controls without family history, previous neuroimaging studies have identified multimodal markers of alterations in the IFG, including gray matter volume and morphology, white matter microstructure, task-based activation, and resting-state connectivity. As a core region of prefrontal regulatory systems, IFG plays an important role in both cognitive and emotion processing, and its alteration may contribute to behavioral deficits in at-risk individuals such as impulsivity, mood liability, inattention, and cognitive dysfunction. Evidence to date suggests that regional IFG structure and activity may contribute to resilience to moderate effects of illness risk, and that IFG functional connectivity may potentially serve as familial risk endophenotype. Although the significance of IFG in BD-risk studies is increasingly realized, few robust and consistent conclusions can yet be drawn from a large number of mixed findings. Large-scale and prospective longitudinal studies using advanced imaging analytic techniques may advance systematic understanding of the role of IFG in genetic vulnerability for BD, and potentially assist in the identification of at-risk individuals for early intervention.
